# JunB: a paradigm for Jun family in immune response and cancer

**DOI:** 10.3389/fcimb.2023.1222265

**Published:** 2023-09-04

**Authors:** Fu-jia Ren, Xiao-yu Cai, Yao Yao, Guo-ying Fang

**Affiliations:** ^1^ Department of Pharmacy, Hangzhou Women’s Hospital, Hangzhou, Zhejiang, China; ^2^ Department of Clinical Pharmacology, Key Laboratory of Clinical Cancer Pharmacology and Toxicology Research of Zhejiang Province, Affiliated Hangzhou First People’s Hospital, Zhejiang University School of Medicine, Hangzhou, China; ^3^ Department of Pharmacy, Women’s Hospital, School of Medicine, Zhejiang University, Hangzhou, China

**Keywords:** AP-1, JunB, immune response, tumorigenesis, tumor microenvironment

## Abstract

Jun B proto-oncogene (JunB) is a crucial member of dimeric activator protein-1 (AP-1) complex, which plays a significant role in various physiological processes, such as placental formation, cardiovascular development, myelopoiesis, angiogenesis, endochondral ossification and epidermis tissue homeostasis. Additionally, it has been reported that JunB has great regulatory functions in innate and adaptive immune responses by regulating the differentiation and cytokine secretion of immune cells including T cells, dendritic cells and macrophages, while also facilitating the effector of neutrophils and natural killer cells. Furthermore, a growing body of studies have shown that JunB is involved in tumorigenesis through regulating cell proliferation, differentiation, senescence and metastasis, particularly affecting the tumor microenvironment through transcriptional promotion or suppression of oncogenes in tumor cells or immune cells. This review summarizes the physiological function of JunB, its immune regulatory function, and its contribution to tumorigenesis, especially focusing on its regulatory mechanisms within tumor-associated immune processes.

## 1 Introduction

The transcription factor AP-1 is a dimeric complex consisting of members from the Jun (c-Jun, JunB, JunD), Fos (c-Fos, FosB, Fra1, Fra2), ATF (activating transcription factor) and MAF (musculoaponeurotic fibrosarcoma) protein families (Eferl and Wagner, 2003). Numerous studies have demonstrated the involvement of AP-1 in diverse biological processes, such as embryo development, tissue homeostasis and inflammation. Noteworthily, JunB can form dimeric AP-1 with Fos and basic leucine zipper ATF-like transcription factor (BATF) family proteins through the basic leucine zipper region (bZIP), subsequently transcriptionally modulating the expression of target genes associated with these aforementioned biological processes(Schorpp-Kistner et al., 1999; Hasan et al., 2017; Singh et al., 2018). For decades, the physiological and pathological functions of JunB have been gradually revealed with the help of transgenic mice, such as JunB^-/-^ Ubi-junB transgenic mice, a mutant that expresses JunB under the control of the human ubiquitin C promoter, in which the lethality of embryos was saved but the expression of JunB was strongly reduced in some adult tissues, even though embryos with JunB gene knockout will die (Schorpp-Kistner et al., 1999; Hess et al., 2003). In addition, JunB has been reported to facilitate fetal angiogenesis and cardiovascular development (Schorpp-Kistner et al., 1999; Yoshitomi et al., 2017), while also playing an essential role in the differentiation and maturation of myeloid cells, as well as for bone development and skin homeostasis (Passegue et al., 2001). Therefore, aberrant JunB expression may give rise to diverse pathological conditions in organisms.

Increasing evidence suggests that JunB plays a crucial role in determining the fate of immune cells and modulating their function through regulating the expression of target genes. Specially, it initiates transcription of key downstream genes such as IL-17A and IL-23R during CD4^+^ T cells differentiation, thereby participating in autoimmune diseases and immune system disorders (Katagiri et al., 2021). For decades, a plethora of studies have consistently demonstrated that the pivotal role of the tumor microenvironment (TME) in driving cancer progression. Apart from neoplastic cells, the TME comprises an abundance of inflammatory cells and non-cellular constituents (Zhao et al., 2021). Interestingly, recent investigations have revealed that JunB influences the infiltration of macrophages and neutrophils within the TME; however, its ultimate effect is paradoxical, suggesting a dual role of JunB in tumorigenesis and development (Arakaki et al., 2016; Wutschka et al., 2021), possibly through regulation of immune cell differentiation and effector functions. While JunB acts as a tumor suppressor in leukemia (Passegue et al., 2001), breast cancer (Wutschka et al., 2021), prostate cancer (Konishi et al., 2008), and epidermal neoplasia (Jin et al., 2011), it behaves as an oncogene in renal cancer (Kanno et al., 2012), ovarian cancer (Xu et al., 2021), multiple myeloma (Fan et al., 2021), and lung cancer (Suphakhong et al., 2022).To date, our understanding of relationship between JunB’s immunomodulatory effects and tumor progression remains limited.

In this review, we explore the physiological function of JunB, its immune regulatory function, and its contribution to tumorigenesis. The potential significance of JunB as a therapeutic target in tumor treatment spans its involvement in inflammation regulation to its contribution to tumor development. Conducting comprehensive investigations into the role of JunB within the TME will contribute a more comprehension of tumor pathogenesis.

## 2 Structure and regulation of JunB

**Figure 1:**
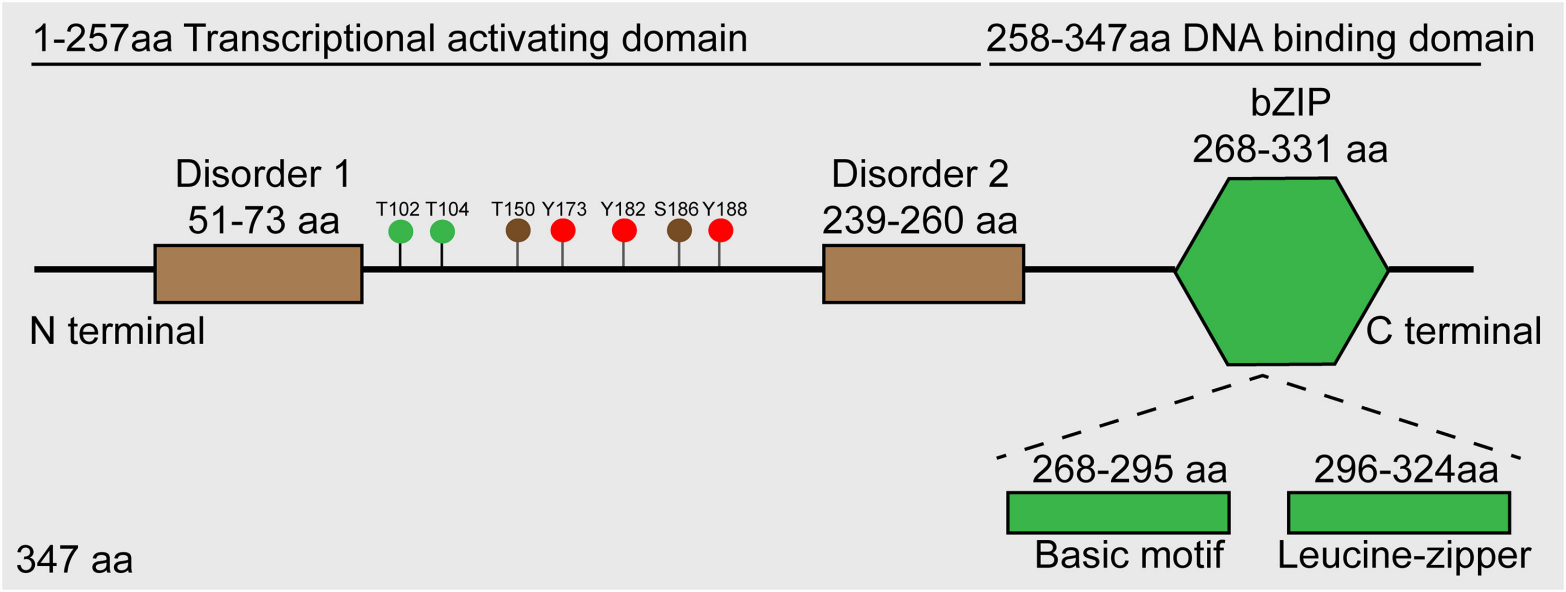
Schematic diagram of JunB protein. Roughly, JunB consists of N-terminal transcriptional activating domain and C-terminal DNA-binding domain. Accurately, there are two disorder regions in transcriptional activating domain, and a bZIP region comprised of basic motif and leucine-zipper regions. The green dots represent sites phosphorylated by JNK, the brown dots represent sites phosphorylated by CDK complexes, and the red dots represent sites phosphorylated by c-Abl.

*JUNB*, a transcription factor located on human chromosome 19p13, encodes a ~35 kDa protein, and its structure includes an α-helical bZIP region, composed of a basic DNA-binding region and a leucine zipper motif characterized with an evenly spaced leucine reside and two disorder regions (Perez-Benavente et al., 2022). According to the functional analysis, JunB is composed of a DNA binding domain at its C terminal and a transcriptional activating domain at its N terminal (Li et al., 2016). Additionally, several post-translational modification sites have been identified, such as phosphorylation sites for JunB (**Figure 1**). Similar to other AP-1 members, JunB can form homodimers or heterodimers with bZIP-containing transcription factors such as c-Fos and BATF family proteins through its bZIP region, thereby regulating the expression of downstream genes. Of note, the hydrophobicity of leucine zipper region in C-terminal of JunB is essential for AP-1 dimer formation (Hasan et al., 2017). So far, there is no evidence to reveal the function of two disorder regions of JunB.

A number of studies suggest that extracellular stimuli, such as growth factors, inflammatory cytokines and stress conditions can induce the expression of JunB transcriptionally. For example, JunB is an immediate target gene in response to TGF-β stimulation, which is formed AP-1 complex with ATF3, repressing the promoter of Id2, an antagonist of TGF-β induced EMT, thus facilitating the TGF-β transcriptional program of EMT and profibrotic processes (Gervasi et al., 2012). In addition, IL-6 is a classical cytokine that induces JunB expression, and JunB also transcriptionally activate the promoter of IL-6 to promote the expression of IL-6 (Gomard et al., 2010; Hasan et al., 2017). External immunostimulatory factors, such as LPS and CpG DNA, significantly induce JunB expression in innate immune cells (Gomard et al., 2010; Fontana et al., 2015b). Moreover, the activation of JNK MAPKs, CDK complexes and c-Abl by these stimuli leads to the phosphorylation of JunB, which promotes the transcriptional activity or destabilization of JunB, thereby modulating inflammation, cell cycle and genome stability in mammalian cells (Li et al., 1999; Farras et al., 2008; Yamaguchi et al., 2015). For example, the phosphorylated JunB at Thr102 and -104 by JNK can promote the synergy between JunB and c-Maf on IL-4 promoter, which is essential for IL-4 expression during T helper cell differentiation. However, phosphorylation of JunB by CDK1/cyclinB1 complexes promotes its degradation in mitosis and late G2 phase, which may be responsible for the transcriptional inactivation of JunB (Bakiri et al., 2000; Farras et al., 2008). It is reported that c-Abl mediated tyrosine phosphorylation of JunB represses its inhibitory effect on the promoter of p21, probably through inhibiting the formation of JunB-c-Fos AP-1 complex, which is involved in the DNA-damage response induced by Adriamycin. It seems that phosphorylation of JunB by c-Abl destabilizes JunB, probably promoting proteasome-mediated degradation. Current studies failed to figure out the cell compartment where c-Abl phosphorylates JunB, since c-Abl and JunB rapidly shuttle between the cytoplasm and nucleus. Whether c-Abl mediated phosphorylation of JunB is a prerequisite for JunB degradation remains to be investigated. Correspondingly, NEDDylation of JunB by itch, a HECT-type NEDDylation ligase, promotes its ubiquitination-dependent degradation, thus impacts its transcriptional activity. Itch NEDDylation also facilitates the degradation of JunB by enhancing the activity of its ubiquitination ligase (Fang et al., 2002; Li et al., 2016). Notably, tyrosine phosphorylation of itch by Src kinase Fyn is vital for itch-mediated the turnover of JunB (Aki et al., 2015). SUMOylation is a reversible post-translational modification that covalently attaches SUMO to the lysine residues of substrate protein, thereby regulating their activity. This process has been identified as a mechanism for transcription factor inactivation. Nevertheless, SUMOylation of JunB in nucleus enhances its transcriptional activity on the promoter of IL-4 and IL-2 in activated CD4^+^ T cells, thus facilitating immune processes mediated by these cytokines. Impairing JunB sumoylation through mutation or utilization of a dominant-negative variant of the SUMO-E2 Ubc-9 significantly attenuated its capacity to transactivate IL-2 and IL-4 reporter genes (Garaude et al., 2008). Overall, phosphorylation, NEDDylation, ubiquitination and SUMOylation may fine-tune the transcriptional activity of JunB through regulating its turnover or influencing dimer formation of AP-1 in physiological conditions, and disruption of this balance may lead to immune disorders, such as accelerating the Th2-dependent allergic response.

**Figure 2:**
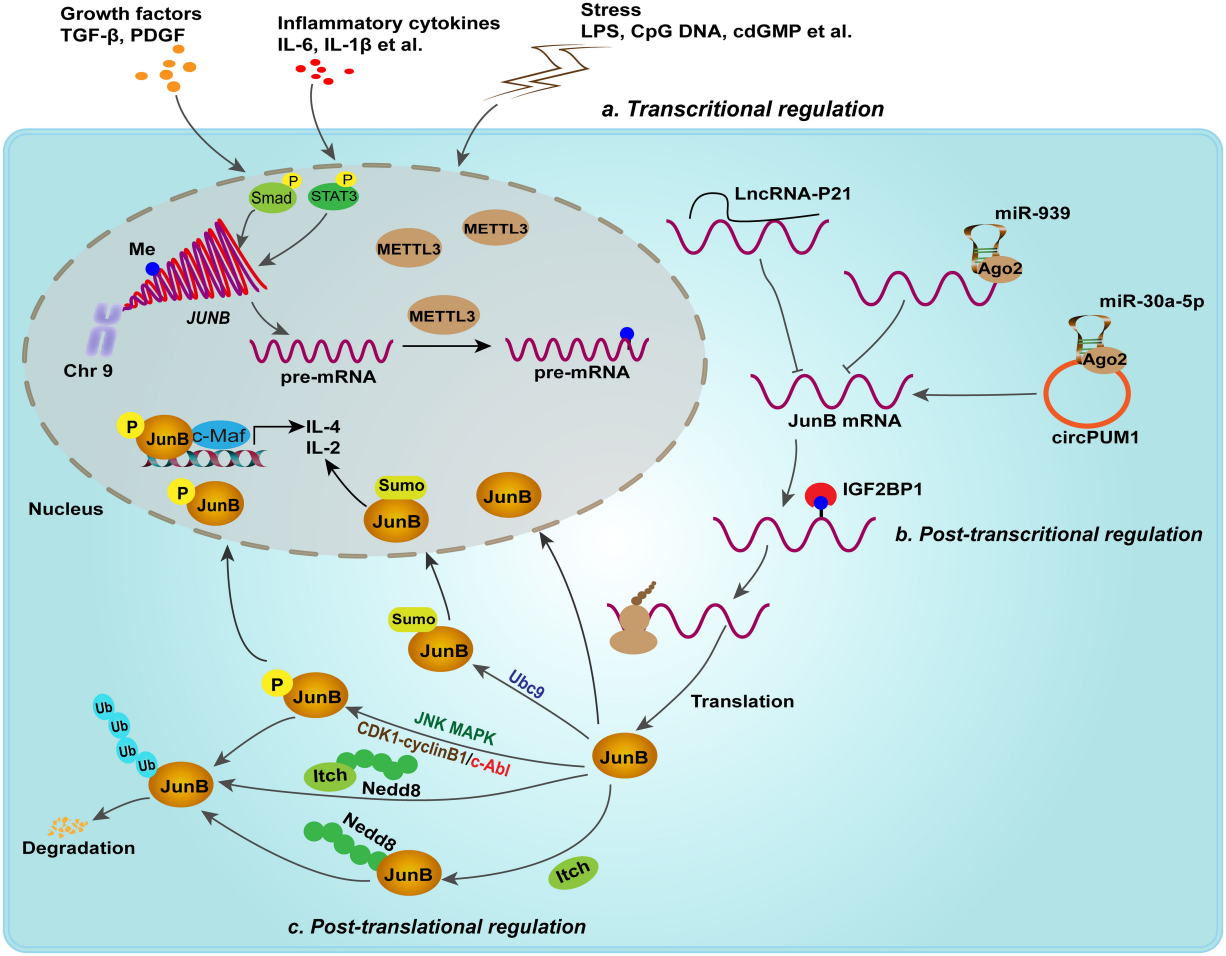
Transcriptional, post-transcriptional and post-translational regulation fine-tune the expression of JunB. In response to growth factors, inflammatory cytokines and external stimuli, JunB is transcriptionally upregulated. For example, p-Smad complex and p-stat3 promote the transcription of *JUNB*. Post-transcriptional regulation including lncRNA, miRNA, circRNA and m6A modification, regulates JunB expression. Moreover, JunB proteins suffer from phosphorylation, NEDDylation, ubiquitination and SUMOylation. Of note, JNK MAPK, CDK complex and c-Abl mediates different phosphorylated sites in JunB, but performing different effects on JunB transcriptionally activities or degradation. In addition, SUMOylation of JunB mediated by Ubc9, a E2 ligase, in nucleus enhances its transcriptional activity on the promoter of IL-4 and IL-2 in activated CD4+ T cells. In context, NEDDylation of JunB mediated by itch or itch itself NEDDylation promotes ubiquitin proteasome-mediated degradation of JunB.

In addition, epigenetic modifications, such as DNA methylation and N6-methyladenosine RNA (m6A) modification, have been reported to regulate the expression of JunB post-transcriptionally. For example, abnormally low expression of JunB in peripheral blood of patients with chronic myeloid leukemia (CML) was found to be attributed to DNA methylation of JunB promoter. Nevertheless, subsequent studies showed that JunB is not a common methylation dependent down-regulated gene (Hoshino et al., 2009; Strathdee et al., 2010). Thus, it is still a controversial issue that needs to be investigated. Recently, emerging studies suggested that m6A modification regulates the expression and stability of JunB in tumorigenesis (Wanna-udom et al., 2020). m6A methyltransferase METTL3 was found to initiate the m6A modification of 3’-UTR of JunB mRNA, subsequently IGF2BP1, one of m6A reader, binds to m6A-modified sites and enhances the stability of JunB mRNA, thereby upregulating the protein expression of JunB, which consequently promotes the EMT of lung cancer cell lines induced by TGF-β1 (Wanna-udom et al., 2020; Suphakhong et al., 2022). Intriguingly, the m6A demethylase FTO was found to preserve the transcription factors of bZIP family, including c-Jun, JunB and C/EBPβ, through its demethylation activity, thereby upregulating the expression of glycolytic genes in tumor cells and facilitating tumor progression (Liu et al., 2021). Mechanical studies indicated that YTHDF2, another m6A reader, promotes the decay of these transcription factors. It seems that different m6A readers define the distinct fate of JunB transcripts and further studies should compare the binding efficacy of m6A readers to JunB transcripts. Non-coding RNAs, such as miR-939 (Garbin et al., 2021), lincRNA-p21 (Yoon et al., 2012) and circPUM1 (Zhu et al., 2021), have also been identified as upstream regulators of JunB in biological processes. Therefore, post-transcriptional and -translational modification may cooperatively regulate the expression level of JunB at homeostasis (**Figure 2**). Upon external stimuli, these epigenetic modifications may be rearranged, resulting in abnormal changes in JunB expression and transcriptional activity. Consequently, the dysregulation of JunB have been found to lead to the occurrence of diverse diseases.

## 3 The physiological role JunB

Previously, it was reported that JunB is involved in the construction of feto-maternal circulatory system, and mice lacking JunB developed embryonic lethality due to the failure of placenta formation and cardiac vasculogenesis (Schorpp-Kistner et al., 1999). Subsequently, more studies have investigated the role of JunB in angiogenesis and vascular development. Knockout of JunB in endothelial cells (ECs) led to embryonic development retardation and death around E10, accompanied by abnormal branches, and vascular system disorder with vasodilation (Licht et al., 2006). It was found that JunB is instantaneously induced in human umbilical vascular endothelial cell (HUVEC) upon VEGF treatment. Intriguingly, knockdown of JunB inhibited migration but did not affect the proliferation of HUVEC, suggesting that VEGF-mediated EC migration is attributed to JunB’s induction (Jia et al., 2016). Everywhere in the body, blood vessels and nerve fibers are generally arranged in close parallel. Yoshitomi et al. demonstrated that neurovascular interactions can promotes the expression of JunB in ECs, thus improving angiogenesis. JunB knockdown in ECs impaired the neurovascular parallel alignment in mouse embryonic skin, reconfirming that JunB is essential for embryonic vascular development (Yoshitomi et al., 2017). In addition, JunB also participates in the outgrowth and differentiation of retinal vascular. Kumar et al. found that VEGFA induces PKCθ phosphorylation in human retinal microvascular endothelial cells, therein JunB is a target of PKCθ. Complete deletion of PKCθ or conditional deletion of JunB in ECs impaired the retinal EC proliferation, tip cell formation and neovascularization induced by hypoxia. Mechanically, JunB could directly bind to the promoter of VEGFR3 in response to VEGFA stimulation, and thereby contribute to VEGFA/VEGFR2-induced retinal neovascularization, which is dependent on PKCθ activation (Kumar et al., 2020). In addition, Licht et al. found that JunB could regulate arterial contraction capacity, cell contractility, and motility in mice through its target myosin regulatory light chain 9, which was confirmed by resistance of Junb-deficient mice to volume-dependent hypertension induced by DOCA-salt and the impairment of isolated arterial contractility (Licht et al., 2010). Therefore, reasonable JunB level is very important for cardiovascular development. However, the pro-angiogenic role of JunB contributes to certain tumor progression, this will be addressed in the following.

In addition, Hess et al. found that endochondral ossification during mid and late gestation also requires JunB, in which JunB regulates the proliferation and function of chondrocytes and osteoblasts (Hess et al., 2003). Notably, the growth of junB^–/–^ Ubi-junB mice was retarded, and showed reduced longitudinal growth (Hess et al., 2003). Moreover, Kenner et al. found that mice lacking JunB in the macrophage-osteoclast lineage develop osteopenia because of cell-autonomous osteoblast and osteoclast defects (Kenner et al., 2011). Smurf1, a ubiquitin ligase, was reported to negatively regulate mesenchymal stem cell proliferation and differentiation into osteoblasts through promoting JunB degradation in a ubiquitin-proteasome pathway (Zhao et al., 2010). Thus, JunB may be a positive regulator of bone growth and remodeling.

Importantly, studies also revealed that JunB participates in myelopoiesis and erythroid differentiation (Passegue et al., 2001; Jacobs-Helber et al., 2002). Though junB^–/–^ Ubi-junB mice escaped from embryonic lethality, they developed a transplantable myeloproliferative disease eventually progressing to blast crisis, resembling human CML (Passegue et al., 2001). Moreover, JunB also maintains the homeostasis of skin immune microenvironment, cutaneous immune cell-microbiota interactions and functional and structural integrity of the epidermo-pilosebaceous unit in the skin (Singh et al., 2018; Uluckan et al., 2019). A variety of pathologically related skin diseases have appeared in mice with specific skin knockout of JunB, including atopic dermatitis (Uluckan et al., 2019), psoriatic disease (Zenz et al., 2005), systemic lupus erythematosus (Pflegerl et al., 2009), ulcerative skin lesions and prolonged inflammation with delayed tissue remodeling (Florin et al., 2006). Therefore, JunB is inextricably linked with immunity. When faced with external stimulation, the improper expression of JunB in organism may cause a series of inflammatory diseases.

## 4 The key role of JunB in immune response

**Figure 3:**
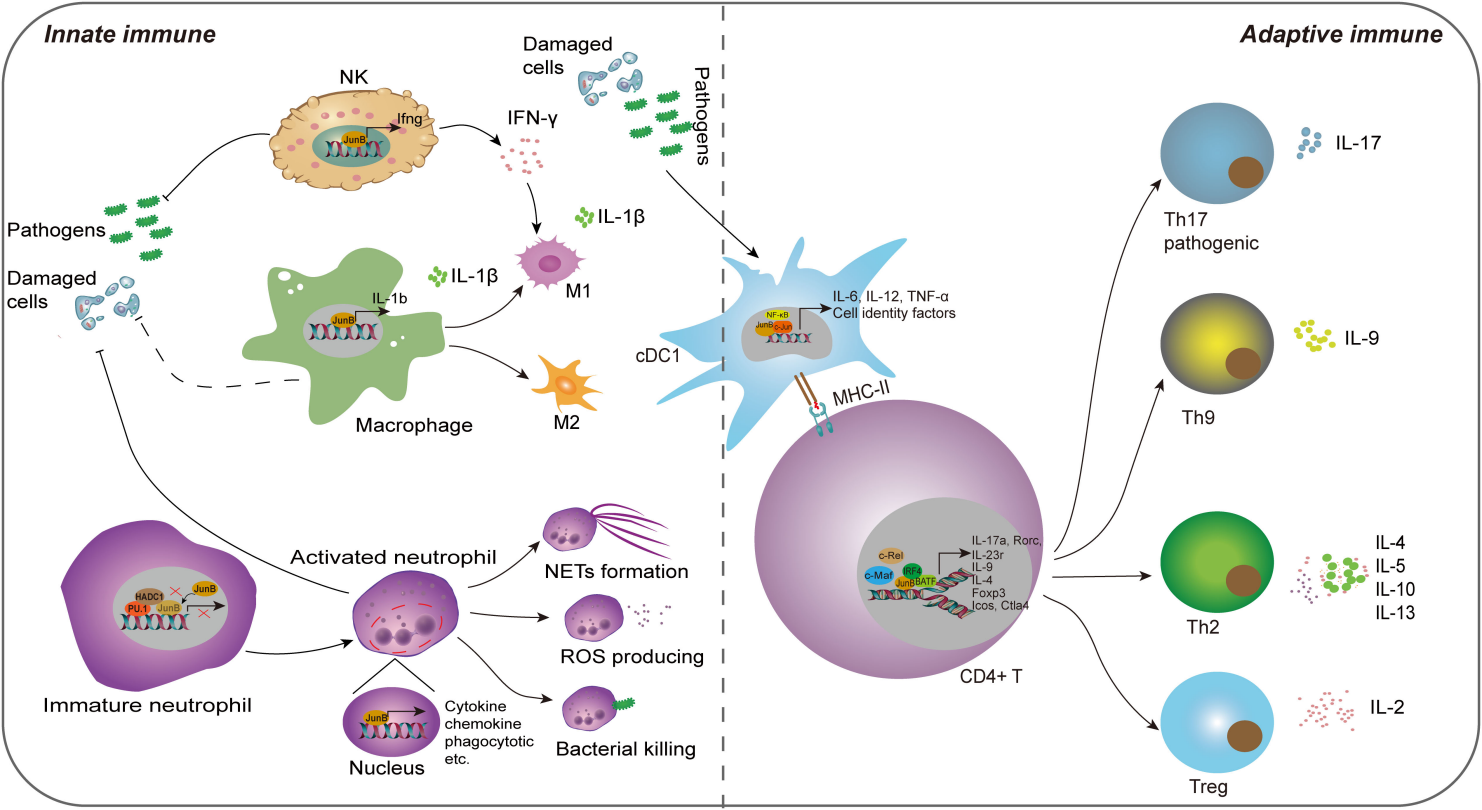
The regulatory role of JunB in innate immunity and adaptive immunity. JunB is upregulated in NK cells, macrophages, neutrophils and cDC1 cells upon external pathogens and self-damaged cells. Subsequently, JunB exerts its pro-inflammatory function through promoting inflammatory cytokine secretion in all these cells, the polarization of macrophages, the effector of neutrophils including NETs formation, ROS production and bacterial killing function through transcriptionally promoting the target gene expression. Specially, the interaction of PU.1, HDAC1 and JunB represses its transcriptional activating role in immature neutrophils. IFN-γ producing from NK cells further promotes M1 macrophage phenotype. In adaptive immunity, JunB promotes the differentiation and activity of CD4+ T cells via cooperating other transcription factors, including c-Maf, c-Rel, NF-κB, BATF and IRF4, thereby enhancing T helper cell activities. Particularly, JunB promotes antigen presentation function of cDC1, which may further activate the adaptive immune system.

It has been reported that AP1 dimeric complex plays an important role in host immune response (Trop-Steinberg and Azar, 2017). As a key part of AP-1, JunB is implicated in the regulation of inflammatory processes, encompassing the modulation of differentiation and functional responses exhibited by myeloid cells. In the following, we will elucidate the regulatory role of JunB in the fate determination, differentiation, and activities of diverse immune cells in innate and adaptive immunity (**Figure 3**). In addition, we have provided a comprehensive overview of JunB in autoimmunity and immunosuppression.

### 4.1 Innate immunity

The innate immune system serves as the first-line of defense against pathogens and self-tissue injuries, eliciting a pro-inflammatory response. Innate immune cells are crucial components that rapidly respond to external or internal stimuli (McComb et al., 2019). Over the past two decades, accumulating evidence has demonstrated JunB’s significant regulatory potential in modulating innate immune cell functions, including promoting pro-inflammatory factor secretion, cell polarization and phagocytosis.

#### 4.1.1 Dendritic cell

Dendritic cells (DCs) represent a subset of innate immune cells that play a pivotal role in initiating antigen-specific immune responses and the maintenance of tolerance(Gardner et al., 2020). Previously, Tiphanie et al. demonstrated the indispensable role of JunB in the production of proinflammatory cytokines IL-6, IL-12 and TNF-α in LPS-stimulated DCs derived from mouse primary bone marrow (Gomard et al., 2010). Mechanically, optimal transcription of these cytokines relies on the synergistic cooperation between JunB and NF-κB, thereby suggesting a regulatory pathway where one transcription factor induces the expression of the other before engaging in cooperative actions (Gomard et al., 2010). Intriguingly, in unstimulated DCs, the JunB gene remains transcriptionally inactive and is organized within a compact chromatin loop, facilitating spatial proximity between its upstream promoter region and downstream enhancer region (Salem et al., 2013). Generally, DCs can be classified into two distinct branches, conventional (cDCs) and plasmacytoid DCs (pDCs) based on their unique transcriptional, functional, and cell surface phenotypes. cDCs can be further subdivided into two subsets, cDC1 which is specialized in cross-presentation, and cDC2 that plays a crucial role in the induction of Th2 and Th17 immunity (Gardner et al., 2020). Notably, the deletion of JunB and c-Jun in DC progenitors remarkably reduced the CD8α cDC1population, resulting in impaired TLR activation and antigen cross-presentation. However, other cDC subsets, such as CD103 cDC1, remained unaffected. These findings suggest that JunB and c-Jun are essential for diversification, function, and identity maintenance of CD8α cDC1 (Novoszel et al., 2021). Therefore, the function of JunB in promoting inflammatory cytokine secretion in DCs is contingent upon the presence of inflammatory milieu, and activated JunB may exert a pivotal regulatory role in cDC1 cell differentiation and immune defense. Given the crucial role of cDCs in anti-tumor immune response (Marciscano and Anandasabapathy, 2021), comprehending the involvement of transcription factors in cDC differentiation and activity is imperative for the development of effective antitumor targets.

#### 4.1.2 Macrophage

Additionally, JunB regulates the polarization and cytokine secretion of macrophages, which are tissue tissue-resident phagocytes of the innate immune system responsible for detecting and restraining infected microorganisms and initiating an immune response. Intriguingly, macrophages with different phenotypes play roles in inflammation and immunoregulation at different stages of the immune response. Traditionally, based on their surface markers or distinct functions, macrophages are divided into M1 (pro-inflammation) and M2 (immunoregulation) macrophages, although subsets of macrophages are complex (Wynn and Vannella, 2016). Initially, the researchers found that JunB was significantly upregulated in macrophages upon stimulation with IL-6, LPS and bovine type I collagen in serum (Oritani et al., 1992; Cho et al., 2002; Frazier-Jessen et al., 2002). Fontana et al. constructed a network regulating macrophages and identified a correlation between JunB and *Il1b*. Myeloid-restricted knockout of JunB led to a remarkable decrease of *Il1b* mRNA and IL-1β protein in BMDMs treated with LPS or LPS plus ATP. Further experiments demonstrated that JunB could regulate macrophage responses stimulated by various immunostimulatory ligands, including poly (I:C), imiquimod, CpG DNA and cyclic-di-GMP (Fontana et al., 2015b). Noteworthily, JunB exerted a significant impact on the polarization of M1 and M2 macrophages, while exhibiting no discernible influence on macrophage proliferation, differentiation, and phagocytosis (Fontana et al., 2015b). However, this study exclusively evaluated the impact of JunB on macrophage polarization *in vitro*. Their subsequent work found that mice deficient in JunB specifically within myeloid cells exhibited resistance to *Plasmodium berghei*-elicited type 1 immune activation, as evidenced by diminished cerebral pathology and improved survival. Additionally, these mice also displayed reduced *Nippostrongylus brasiliensis*-elicited type 2 activation, characterized by decreased cytokine secretion and eosinophil recruitment alongside increased parasite burden(Fontana et al., 2015a). In general, these studies suggest that JunB plays a crucial role in macrophage-mediated immune responses. With the development of single-cell RNA sequencing (scRNA-seq) technology, more macrophage subtypes have been identified. Recently, a scRNA-seq study conducted on peripheral immune cells of patients with systemic sclerosis (SSc) revealed a novel inflammatory gene module originating from CD16^+^ monocytes, which included KLF10, PLAUR, JUNB and JUND; this module displayed the greatest discrimination compared to healthy control group(Kobayashi et al., 2021). It is intriguing to consider whether JunB plays a role in determining the fate of additional macrophage subtypes. Furthermore, the observation that JunB modulates macrophage activity within the TME by transcriptionally regulating chemokine secretion from tumor cells provides valuable insight, as M2 macrophages infiltration exacerbates solid tumor progression (Arakaki et al., 2016; Cassetta and Pollard, 2018). This suggests that JunB may serve as a crucial regulator in the macrophage polarization within the tumor microenvironment (TME).

#### 4.1.3 Neutrophil

Similar to macrophages, neutrophils are skilled phagocytes that digest and destroy invading pathogens. Besides, neutrophils play an immunoregulatory role in both the innate and adaptive systems due to their capacity in producing pro-inflammatory or immune-suppressive cytokines. However, when over-activated, their strong immune response can cause collateral damage, which may lead to the occurrence of immune diseases (Liew and Kubes, 2019). Recent studies have unveiled that JunB functions as a lineage-determining transcriptional factor that plays a crucial role in promoting neutrophil cell survival and establishing their effector function repertoire through transcriptionally accessible. Upon activation, JunB becomes activated and subsequently occupies the promoter regions of target genes, thereby facilitating the effector of neutrophils (Khoyratty et al., 2021). Intriguingly, it has been reported that PU.1 (encoded by *Spi1* gene) recruits HDAC1 to repress the expression of immune-related genes, leading to a reduction in AP-1-binding motif accessibility within enhancers. Notably, among all AP-1 transcription factors present in activated neutrophils, JunB is found to be the most abundant transcript; however, PU.1 prevents JunB from accessing to enhancers in immature neutrophils (Fischer et al., 2019). Thus, the interaction between PU.1, JunB and HADC1 plays a crucial role in regulating neutrophil activities. Subsequently, Khoyratty et al. conducted transcriptional and chromatin analyses of neutrophils during acute inflammation and demonstrated that JunB is capable of driving various effector responses in neutrophils, such as enhancing reactive oxygen species (ROS) production, bacterial killing and NET formation (Khoyratty et al., 2021). In the mouse model of acute myocardial infarction, JunB knockout in neutrophils attenuated pathological inflammation (Khoyratty et al., 2021). Overall, under normal conditions, PU.1 controls JunB; however, during inflammation, the repressive effect of PU.1 on JunB is abrogated, leading to activation of JunB and subsequent enhancement of transcriptional activity that promotes neutrophil-mediated immune processes. Nevertheless, stromal loss of JunB was found to promote the infiltration of neutrophil in tumor metastasis (Fischer et al., 2019; Wutschka et al., 2021). The bidirectional role of JunB in immune regulation is evident as its regulatory effects on neutrophils may exhibit reversibility in other inflammatory diseases and tumor states.

#### 4.1.4 Natural killer cell

In innate immune system, natural killer (NK) cells are effector cells that perform cytolysis and cytokine-producing function. Their specialized receptors, such as NKG2D, monitor infected neighboring cells by recognizing the absence of cell surface MHC (McComb et al., 2019). The binding of NKG2D (receptor) with ligand RAE-1 plays an important role in the anti-tumor immune response mediated by NK cell, γδ^+^ T and CD8^+^ T cells. Nausch et al. found that JunB knockdown enhanced cell surface expression of RAE-1 in mouse embryonic cell lines, which was helpful to enhance the killing ability of NK cells to JunB-deficient cells and IFN-γ production through NKG2D(Nausch et al., 2006). This study suggests that due to loss of JunB, the upregulation of RAE-1 can alarm immune cells to tumors or abnormal stressed cells. Inconsistently, Wang et al. found that JunB is a cofactor required for Smad4-mediated maturation, homeostasis and anti-tumor effects of NK cells by potentiating expression of granzyme B (Wang et al., 2018). In addition, Thomsen et al. found that JunB can directly bind to the promoter of IFN-γ in NK/NKT cells, promoting transcription and production of IFN-γ, and thereby aggravating acute hepatitis (Thomsen et al., 2013). These studies also suggest a biphasic role for JunB in tumor and NK cells. However, the current study is not clear whether JunB promotes or inhibits the effect of NK cells in the tumor microenvironment, and more studies are needed to prove it.

### 4.2 Adaptive immunity

Adaptive immunity selects the most suitable immune receptors to target infectious antigens, which is one of its most powerful elements. In fact, the delineation of JunB’s role in innate and adaptive immunity is not rigid, as innate immunity serves as the foundation for adaptive immunity. Particularly, JunB promotes differentiation of cDC1 cells, promotes their antigen presentation, which may further activate the adaptive immune system (Novoszel et al., 2021). Most studies have focused on the regulation of T cell-mediated immune responses by JunB. T cells can be generally divided into two groups based on the CD4^+^ or CD8^+^ receptors on the cell surface (McComb et al., 2019). It is well known that CD4^+^ T helper (Th) cells are a group of cytokine-producing cells with high heterogeneity. The effector Th cells play a key role in coordinating the immune response elicited by different infections, and participate in the occurrence and development of various autoimmune diseases, such as psoriasis, allergy and asthma (Raphael et al., 2015). Throughout research history, JunB is considered as an important regulator of the fate of CD4^+^ T cells. Li et al. first found that JunB, instead of c-Jun or JunD, was selectively induced in Th2, but not in Th1 during T cells differentiation (Li et al., 1999). Mechanically, JNK MAPK mediated JunB phosphorylation at Thr102 and -104 directly binds to the promoter region of IL-4, and cooperates with c-Maf to promote the expression of Th2-restricted IL-4 (Li et al., 1999). In innate immune system, IL-4 promotes the maturation of DCs and the polarization of M2 macrophages, thus JunB also indirectly affects innate immune responses. In addition, JunB also enhanced the expression of IL-5 and IL-10 (Hartenstein et al., 2002). As these cytokines produced by Th2 cells play a vital role in humoral immunity and the allergic reaction, JunB may be a key mediator in regulating their expression in autoimmune and allergic diseases. Itch is suppressed in itchy mice. Intriguingly, JunB was reported to be upregulated abnormally in Itch-deficient mice, and these mice developed severe immune disorders and constant skin itching (Fang et al., 2002). The reason is that JunB undergoes Itch-mediated ubiquitination degradation in normal mice, and Itch deficiency causes abnormal accumulation of JunB, thus leading to the production of Th2-dependent allergic factors, including IL-4, IL-5, IgG1 and IgE (Fang et al., 2002). Consistently, JunB knockout in mouse CD4^+^ T cells led to impaired differentiation of Th2 cell, accompanied by the imbalance of IL-4 and IL-5, and these mice displayed improved allergen-induced airway inflammation (Hartenstein et al., 2002). Chen et al. reported that TCR stimulation (anti-CD3/CD28) promoted USP38 in allergic asthma. In turn, USP38 improved the protein stability of JunB. Functionally, USP38 was necessary for production of Th2 cytokines (IL-4, IL-5 and IL-13) induced by TCR, and mice with USP38 knockout were refractory to asthma induced by OVA or HDM (Chen et al., 2018). Most recently, Hsieh et al. found that JunB plays a key role in the clonal proliferation of a variety of T helper cells by inhibiting their apoptosis (Hsieh et al., 2022). Therefore, JunB is essential for the survival and differentiation of Th2 cells and Th2-mediated autoimmune diseases.

Nevertheless, increasing studies report that JunB is also involved in differentiation of regulatory T (Treg) and Treg-mediated immune homeostasis (Koizumi et al., 2018). It is widely known that immune homeostasis is critical for human health. Effector Treg cells, differentiated from Foxp3-expressing CD4^+^ Treg cells, play an important role in immune homeostasis by inhibiting various anti-self or innocuous antigens. Son et al. reported that JunB and c-Rel synergistically promote the expression of Foxp3 through binding its promoter in the process of Treg differentiation induced by TCR signal and TGF-β/IL-2 (Son et al., 2011). Notably, JunB was reported to upregulate in effector Treg cells and play an essential role in eTreg-mediated immune homeostasis, and mice with JunB knockout specifically in Treg cells were reported to develop multi-organ autoimmunity, accompanied by abnormal activation of Th cells and cytokines production, including IL-4, IL-13, IFN-γ and IL-17A (Koizumi et al., 2018; Wheaton and Ciofani, 2020). Mechanically, JunB promoted the accumulation of IRF4 on a series of IRF4 target, including those located near *Icos* and *Ctla* 4 (Koizumi et al., 2018). In addition, due to the damage of Treg differentiation, the conditional knockout of JunB in mouse CD4^+^ T cell is more susceptible to colitis induced by dextran sulfate sodium (DSS), and deficiency of JunB *in vitro* CD4^+^ T cells resulted in the failure of IL-2-induced Treg cell differentiation (Katagiri et al., 2019). Therefore, JunB is a key regulator of Treg differentiation and effect program dependent on different mechanisms and Treg-mediated immune homeostasis. To keep immune tolerance, the activity of effector T cell must be monitored by Tregs. Wu et al. found that Tregs sense effector T cell by coordinating JunB expression, and JunB deficiency weakens Treg identity and leads to the uncontrolled secretion of inflammatory cytokines and spontaneous inflammation dependent on T-bet, a T-box transcription factor (Wu et al., 2019). In a mouse model of melanoma, deficiency of JunB in Tregs could release anti-tumor potential of CD4^+^ T cells, suggesting JunB enhances Treg-mediated immune tolerance in tumorigenesis (Wu et al., 2019).

In addition to coordinating genes that regulate the differentiation of Th2 and Treg cells, JunB also activates the expression of specific genes of the Th17 lineage and promotes Th17 cell identity (Carr et al., 2017). At present, IL-17-producing CD4^+^ T cells, also known as Th17 cells, have been classified into two groups, pathogenic and non-pathogenic Th17 cells separately, which perform different biological functions. Hasan et al. found that JunB induced by IL-6 is essential for expression of RORgt and IL-23 receptor by facilitating DNA binding of BATF at the Rorc locus in IL-23-dependent pathogenic Th17 cells, but not in TGF-β-dependent non-pathogenic Th17 cells (Hasan et al., 2017). Contrary to DSS-induced colitis mentioned above, mice lacking JunB in CD4^+^ T cells were resistant to Th17-mediated autoimmune encephalomyelitis and colitis. Moreover, JunB-deficient CD4^+^ T cells could develop into various T helper subsets except Th17 *in vitro* (Hasan et al., 2017).

JunB was also reported to cooperate with BATF to promote the specificity of BATF-dependent cytokine induction in other Th subsets. Th9 cells, the recently defined Th subsets, show higher expression of Jun family member, including JunB and c-Jun, but not JunD compared to Th17 cells. Silencing JunB or c-Jun could reduce IL-9 expression, but it does not affect *Batf* or *Irf4* expression in IL-9 cells, suggesting JunB and c-Jun are required for Th9 differentiation. Mechanical experiments showed that IRF4 stabilizes the BATF-JunB heterodimer of IL-9 gene in Th9 cells (Fu et al., 2019). Overall, these studies indicate that JunB plays an important role in Th and Treg differentiation and adaptive immune response mediated by CD4^+^ T. However, no research has been reported on which type of T-cell JunB has the strongest promoting effect as a cooperative promoter. It seems that JunB is not specific enough for T cell subsets differentiation, which may be related to JunB activation and upstream regulation in different inflammatory disease states.

Given its crucial role in differentiation and cytokine-producing of immune cells, JunB may be a potential regulator for immune disorders. Noteworthily, JunB promotes the progression of Th2-mediated autoimmune diseases, such as asthma (Hartenstein et al., 2002) and skin allergy (Fang et al., 2002), and Th17-mediated autoimmune encephalomyelitis and colitis (Carr et al., 2017). Furthermore, JunB is a cell marker for CD16^+^ monocytes in SSc, suggesting JunB may affect the differentiation of this cell group during the progression of SSc (Kobayashi et al., 2021). JunB also promotes Treg-mediated immunosuppression, and the absence of JunB leads to inflammatory disorders, such as inflammatory bowel diseases (Katagiri et al., 2019). In addition, the inhibitory effect of JunB on neutrophils in tumors also reflects its immunosuppressive effect through transcriptional control rather than activation of target genes (Wutschka et al., 2021). In contrast, JunB-mediated immune tolerance of Tregs promotes immune escape of melanoma cells(Wu, et al., 2019). Therefore, the regulation of JunB on the immune microenvironment depends on its preferential transcriptional regulation of the immune effectors across different cells, thereby potentially playing significant roles in autoimmunity and immunosuppression.

## 5 Role of JunB in tumorigenesis

**Figure 4:**
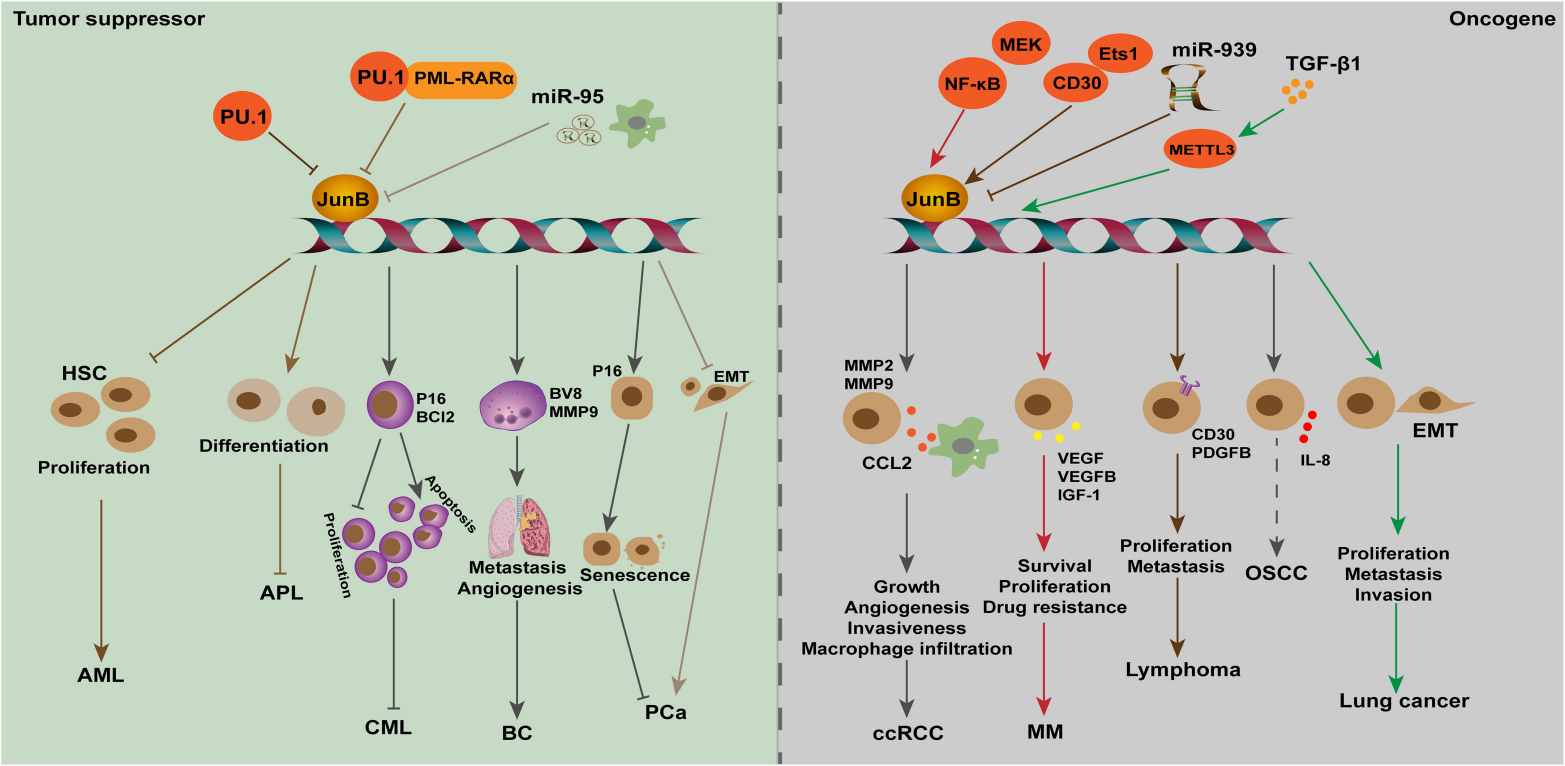
The dual role of JunB in tumorigenesis is evident across various cancer types. In leukemia (AML, APL, and CML), breast cancer, and PCa, JunB exerts a tumor suppressive effect by inhibiting cell proliferation, differentiation and metastasis, and promoting senescence processes. Transcriptional accessibility of JunB in AML is regulated by PU.1 and PU.1/PML-RARα. Conversely, in ccRCC, MM, lymphoma, OSCC and lung cancer, JunB plays an oncogenic role through the promotion of CCL2 secretion as well as enhancing VEGF-, IGF1-, and IL-8-mediated signaling pathways involved in tumor progression. Additionally, it influences gene transcription during EMT process or facilitates surface receptor abundance on tumor cells. Notably, NK-κB activation along with CD30 enhances the expression of JunB transcriptionally and promotes its transcriptional activities on target genes. In response to TGF-β1 stimulation, METTL3 facilitates the upregulation of JunB by promoting m6A methylation in JunB transcripts, thereby contributing to the EMT process in lung cancer cells. Additionally, miR-939 acts as an inhibitor of JunB in lymphoma.

Increasing evidence suggests that JunB is usually dyregulated in cancer and can perform tumor suppressive or oncogenic role depending on the cancer entity. Generally, JunB regulates cell cycle, differentiation, senescence, metabolism, and metastasis of tumor cells to affect tumor progression (Konishi et al., 2008; Santaguida et al., 2009; Liu et al., 2021; Wutschka et al., 2021; Perez-Benavente et al., 2022). Inflammation is closely associated with tumorigenesis and development. As a potent regulator of immune processes, the role of JunB in the TME should not be ignored, which will benefit the development of cancer immunotherapy targets. Noteworthily, recent studies revealed that JunB is enriched in stromal cells (immune cells, endothelial cells and fibroblasts) in TME, suggesting that JunB may be a critical regulator in TME. In the following, we will address the role and regulatory mechanism of JunB in different cancers (**Figure 4**).

### 5.1 Tumor suppressor

#### 5.1.1 Leukemia

Leukemia is a series of malignant diseases of blood and bone marrow that threaten human life. Genetic abnormalities are widely known to be associated with leukemia occurrence (Juliusson and Hough, 2016). Notably, patients with acute or chronic myeloid leukemia presented low expression levels of JunB, increasing studies suggested JunB affect cell cycle and differentiation through transcriptionally activating essential genes. Transcription factor PU.1 is necessary for granulocyte monocyte differentiation during normal hematopoiesis. Knocking out terminal enhancer of PU.1 reduces the expression level of PU.1 in bone marrow by 80%, resulting in the occurrence of acute myeloid leukemia (AML) in mice (Rosenbauer et al., 2004; Rosenbauer et al., 2005). On this basis, Steidl et al. found that JunB was significantly down-regulated in PU.1 knockdown preleukemia hematopoietic stem cells. Lentivirus-mediated JunB repair can make NOD-SCID mice lose their self-renewal ability and inhibit the progress of leukemia. In addition, correlation between the down-regulation of PU.1 and JunB was confirmed by examination of AML patients. The decrease of JunB expression might be one of the causes of malignant self-renewal of HSC in AML patients (Somervaille and Cleary, 2006; Steidl et al., 2006). Deletion of two orphan nuclear receptors, Nr4a3 and Nr4a1, also led to the occurrence of AML. In Nr4a3^-/-^Nr4a1^-/-^ mice, the expression of PU.1 was not affected, but the transcripts of c-Jun and JunB were significantly down-regulated, indicating that the downregulation of AP-1 transcription factors may contribute to the development of AML in these mice (Mullican et al., 2007). Santaguida et al. found that JunB inactivation disturbs the regulation of cell cycle and increases the proliferation of long-term repopulating HSCs (LT-HSCs) without affecting its self-renewal *in vivo*. JunB frequently controls the proliferation of LT-HSCs and limits the rate of myeloid progenitor cells production by maintaining proper response to Notch and TGF-β signals, partly through the transcriptional regulation of these two pathways, which are important mediators of Hes1. The deletion of JunB gene destroyed the stability of the network of genes and pathways that regulate the fate of HSC, resulting in abnormal expansion of myeloid progenitor cells, which promotes the occurrence of myeloid malignant tumors (Guzman and Jordan, 2009; Santaguida et al., 2009). Fiskus et al. found that the inhibitory effect of panobinostat (a pan-HADC inhibitor) combined with dexitabine on human AML cells was stronger than that of one of them. Mechanical studies showed dexitabine induced demethylation of JunB promoter, and panobinostat treatment mediated chromatin change of JunB promoter to increase JunB level, both of them have synergistic effects (Fiskus et al., 2009). In acute promyelocytic leukemia (APL), S-phase kinase-associated protein 2 (Skp2) can inhibit autophagy by reducing the expression of lncRNA HOTAIRM1 and the inactivation of GSK3β, and then inhibits the ubiquitination and degradation of PML-RARα, thus inhibiting the transcriptional activation of JunB through the PU.1/PML-RARα transcriptional complex, and ultimately prevents cell differentiation and promotes the progress of APL. When NB4 cells were treated with ATRA and GSK3β inhibitors, the expression of Skp2 was down-regulated, while the expression of JunB was significantly induced by accelerating the degradation of PML-RARα, which contributed to the eradication of APL(Dan et al., 2022). JunB was identified as the target gene of miR-149^*^, an oncogenic miRNA, in T-cell acute lymphoblastic leukemia, suggesting that JunB may be a tumor suppressor (Fan et al., 2016). Therefore, restoration of JunB in human acute leukemias may improve disease progression.

Passegue et al. found that lack of JunB in mice can lead to myeloproliferative diseases, similar to human CML (Passegue et al., 2001). Subsequently, yang et al. found that the expression of JunB gene was significantly down-regulated in both CML cells and patients. Low expression of JunB was associated with the clinical stage of the patient, that is, in blast crisis it is less than chronic phase which is less than normal. Mechanism studies have shown that the CpG site in the JunB promoter region of CML patients is highly methylated, and 5-aza-2-deoxycytidine demethylation can partially restore the expression level of JunB, indicating that JunB methylation may be a cause leading to CML (Yang et al., 2003). However, Hoshino et al. found that the down-regulation of JunB expression in advanced CML is not achieved through DNA methylation, which was suggested by the fact that JunB methylation was not observed in all CML cell lines studied, and only 3% of CML patients showed JunB methylation (Hoshino et al., 2009). Therefore, the cause of abnormally low level of JunB in CML needs further investigation, and post-translational regulation should be taken into consideration. Liu et al. used JunB as a detection index to evaluate the efficacy of CML patients after receiving imatinib treatment, and found that increased expression of JunB combined with decreased expression of BCR-ABL is a good predictor of clinical prognosis in patients with CML after receiving imatinib treatment (Liu et al., 2006). However, more clinical studies and long-term follow-up are needed, and the intrinsic mechanisms of JunB in leukemia needs to be revealed.

#### 5.1.2 Breast cancer

Female breast cancer (BC) has now surpassed lung cancer, becoming the leading cause of global cancer incidence in 2020. It is estimated that there are 2.3 million new cases, accounting for 11.7% of all cancer cases (Sung et al., 2021). Organ metastasis is one of the leading causes of high mortality in BC. Recent studies shows that bone, lung, liver and brain are generally regarded as the main targets for BC metastasis (Liang et al., 2020). In 2013, Kharman-Biz et al. revealed the expression of JunB was negatively correlated with tumor stage and lymph node status, speculating that increased JunB may be related to a less aggressive phenotype and indicating a tumor suppressive role of JunB in BC (Kharman-Biz et al., 2013). Intriguingly, the expression of JunB is significantly upregulated in patients with inflammatory breast cancer compared to those with non-inflammatory breast cancer (Bieche et al., 2004), suggesting that JunB may play a regulatory role in the underlying inflammatory processes in. The TME is composed of all the non-cancerous cells in the tumor, including fibroblasts, endothelial cells, neurons, adipocytes, adaptive, and innate immune cells, and the non-cellular components, including cytokines, chemokines, growth factors, extracellular vesicles and extracellular matrix (ECM)(Xiao and Yu, 2021). Communications between non-cancer cells and cancer cells in TME are critical regulators of tumor initiation, growth and metastasis (Zhao et al., 2021). Recently, Schulz et al. found that JunB was highly expressed in stromal cells of human breast cancer, such as endothelial cells, cancer associated fibroblasts (CAFs) and immune cells. Stromal loss of JunB in mice led to increased tumor metastasis to lung. In addition, an increase in the density of pulmonary vessels was observed in JunB knockout mice. These results remind us of the key regulatory functions of JunB in neovascularization, angiogenesis and vascular system. It is known that an aberrant remodeled tumor vessel and frequently leakier vasculature enhance the intravasation and extravasation of disseminated tumor cells(Schulz et al., 2019). Nevertheless, the tumor blood and lymphatic vascular density and integrity were not influenced upon JunB deficiency. Of note, lack of JunB promoted the recruitment of myeloid cells at the early metastatic stage. Further experiments concluded that a significant increase of neutrophils in circulation is the main reason for distant metastasis induced by JunB deficiency. BV8 and MMP9, two proteins that promote angiogenesis and tissue remodeling, were upregulated in these neutrophils in a JunB-dependent manner, suggesting that neutrophils with JunB loss facilitates angiogenesis and vascular remodeling in the pre-metastatic lungs (Wutschka et al., 2021). It can be concluded that JunB performs a tumor suppressive role in breast cancer through regulating the performance of neutrophils in the TME. However, specific knockout of JunB in CAFs, a major cell group in the TME, had no effect on distant metastasis of breast cancer cells, indicating that an altered secretome of JunB-KO CAFs is not the metastatic driver although JunB regulates the proliferation, differentiation and senescence of fibroblasts (Papaioannou et al., 2018; Maity et al., 2021; Wutschka et al., 2021). In addition, the regulatory role of JunB in T cells was also excluded since T cell infiltration is not obvious in the early metastatic lungs (Wutschka et al., 2021), although JunB is recognized as a fate determiner of T cells.

#### 5.1.3 Prostate cancer

According to GLOBOCAN in 2020, prostate cancer (PCa) is the second most common cancer among men worldwide, and its incidence may still rise (Sung et al., 2021). Transient amplifying cells (TACs) are a group of basal cell populations in the prostate, and are considered as the origin of PCa (Schalken and van Leenders, 2003). In recent years, JunB was found to decrease during PCa development, which promoted proliferation, invasion and senescence of PCa cells, as well as remodeled the TME. Konishi et al. reported that JunB expression closely paralleled the levels of p16 expression in both pre-senescent and post-senescent TACs. Further experiments indicated that JunB is an important upstream regulator of p16, which contributes to cell senescence and thus prevents the malignant transformation of TAC (Konishi et al., 2008). Later, one study conducted by Thomsen et al. reconfirmed a suppressive role of JunB in PCa. Mechanically, they found that JunB can prevent invasive PCa cells in Pten-deficient mice, and the absence of JunB leads to increased proliferation and decreased senescence of TACs. In addition, loss of JunB in prostate epithelium of Pten-deficient mice led to the change of surrounding stroma, as suggested by the increase of S100A8, S100A9 and SPP1, proteins related to aggressive PCa, especially in monocytes/macrophages although didn’t influence the number of infiltrating immune cells (Thomsen et al., 2015). JunB may regulate the cross-talk between stromal cells and TACs, thereby repressing PCa. Noteworthily, a recent study by Guan et al. found that miR-95, which was delivered by exosomes from tumor-associated macrophages, promoted proliferation, invasion, and EMT of PCa cell by targeting JunB (Guan et al., 2020). Therefore, JunB possibly inhibits the occurrence of PCa by impacting the expression of key non-cellular constituents in TME. In addition, since JunB is an important determinant of immune cells fate and regulates the secretion of inflammatory cytokines, it is an interesting question whether JunB can promote the anti-tumor effect of immune cells in PCa.

### 5.2 Oncogene

#### 5.2.1 Renal cell carcinoma

Renal cell carcinoma (RCC) is one of the most fatal diseases of the genitourinary system, among which clear cell RCC (ccRCC) accounts for 75-80% of total cases (Ahluwalia et al., 2021). At the molecular level, the loss of tumor suppressor gene von Hippel-Lindau (VHL) function on chromosome 3p25 led to the occurrence of ccRCC. Kanno and co-workers found that JunB increased in VHL-defective ccRCC samples, and JunB knockdown inhibited invasiveness of VHL-null ccRCC cells by controlling the expression of MMP2, MMP9 and chemokine (C-C motif) ligand-2 (CCL2). *In vivo* xenograft tumor results demonstrated that inhibition of JunB could repress tumor growth and angiogenesis, suggesting that JunB may be an oncogene in ccRCC (Kanno et al., 2012). Further studies suggested that CCL2, the downstream effector of JunB, promotes ccRCC through promoting tumor angiogenesis and recruiting macrophages. Thus, JunB may regulate the TME in ccRCC, but whether JunB directly control the stromal cells remains to be investigated (Arakaki et al., 2016; Zhang et al., 2021). However, the upstream regulatory factors that promote the abnormal expression of JunB in ccRCC have not been revealed. Given that growth factors and inflammation factors have been shown to stimulate the expression of JunB, it raises the question whether non-cellular constituents within the TME may also influence JunB expression in ccRCC.

#### 5.2.2 Multiple myeloma

Multiple myeloma (MM) is the second most common hematological malignancy, characterized by excessive clonal proliferation of malignant plasma cells in the bone marrow (BM), renal disease, immune deficiency, and osteolytic bone lesions, which brings economic burden to human beings (Fan et al., 2021). Increasing BM angiogenesis accelerates disease progression in MM patients and correlates poor prognosis. Recently, Fan et al. found that JunB was significantly induced in MM cells when co-cultured with bone marrow stromal cells (BMSCs), while other AP-1 members, such as c-Jun, JunD, c-Fos, and c-Maf, were moderately upregulated or undetectable (Fan et al., 2017). Further investigations indicated the indispensable role of JunB in MM, including promoting cell survival, proliferation and drug resistance, as well as activating the transcription of pro-angiogenic factors (AFs), such as VEGF, VEGFB, and IGF1, and eventually leading to angiogenesis of bone marrow in MM models (Fan et al., 2017; Fan et al., 2021). Notably, the abnormal upregulation of JunB was elicited by stroma, particularly IL-6, but not hypoxia, which is MEK/MAPK- and NF-κB-dependent but Ras-independent mediator for the transcription of abovementioned AFs (Fan et al., 2021). Therefore, TME influences the expression of JunB in tumor cells, which plays a vital role in promoting BM angiogenesis during MM development. However, the intrinsic molecular mechanism of JunB upregulation is still unclear, and whether other stimuli, such as stiffness of ECM in the TME, promotes MM growth remains to be investigated. In addition, proper expression of JunB is essential to maintain a pro-angiogenic role of endothelial cells at homeostasis *in vivo*. In malignancies, such as MM, drug design for JunB will help to develop new strategies to slow down the progress of cancer.

#### 5.2.3 Lymphoma

A growing number of studies found that JunB is a potential oncogene in lymphoma, including Hodgkin lymphoma (HL) and anaplastic large cell lymphoma (ALCL). HL is most frequently seen in young adults, and is classified into classical HL, an aggressive phenotype, and nodular lymphocyte-predominant HL, an indolent phenotype, according to histomorphology (Shanbhag and Ambinder, 2018). ALCL is a rare aggressive peripheral/mature T-cell lymphoma in children and young adults, and is classified into ALK-positive (ALK+) and ALK-negative (ALK-) ALCL (Leventaki et al., 2020). Aberrant upregulation of c-Jun, JunB and CD30 is a hallmark of tumor cells in HL and ALCL. In 2002, Mathas et al. found that ectopic expression of c-Jun and JunB promoted proliferation of HL cells *in vitro*. Differently, upregulation of c-Jun is mediated by an autoregulatory pattern while JunB is dependent on NF-κB activation in HRS cells (Mathas et al., 2002). Besides, knockdown of c-Jun or JunB inhibited the growth of classical HL cell lines through augmenting G1 phase arrestation (Mathas et al., 2002). This role of JunB instead of c-Jun was confirmed in ALK-positive ALCL (Zhang et al., 2018), which indicated that c-Jun and JunB had similar roles in HL, but different roles in ALCL. Nevertheless, Watanabe et al. reported that in ALCL and HRS cells, CD30 induced JunB expression by activating ERK1/2-MAPK signals, which is not nuclear NF-κB dependent way. In parallel, JunB could bind to the unmethylated promoter of CD30 through the AP-1 site in ALCL, thereby contributing to high expression of CD30 (Watanabe et al., 2005; Watanabe et al., 2008). ALCLs are frequently associated with chromosomal translocations, leading to abnormal expression of nucleophosmin-anaplastic lymphoma kinase (NPM-ALK). Subsequently, they found that E26 transformation-specific-1 (Ets-1) promotes the activity of JunB promoter, which depended on CD30 or NPM-ALK-ERK1/2 MAPK pathway in HL and ALCL(Watanabe et al., 2012). Laimer et al. found that in NPM-ALK-induced mouse lymphoma models, c-Jun and JunB enhanced lymphoma development and tumor metastasis by transcriptionally regulating platelet-derived growth factor receptor B (*PDGFRB*) expression. Moreover, their evidence showed that therapeutic inhibition of *PDGFRB* significantly prolonged the survival time of NPM-ALK transgenic mice, and promoted the efficacy of ALK-specific inhibitors in transplanted NPM-ALK tumors (Laimer et al., 2012). In pediatric ALCL, it was reported that miR-939 regulates *PDGFRB* expression through targeting JunB. Although c-Jun is also important for regulating *PDGFRB* transcription in ALCL, overexpression of miR-939 does not affect c-Jun expression (Garbin et al., 2021). Therefore, JunB is a crucial transcription factor in HL and ALCL, and it is necessary to explore new drugs targeting JunB to inhibit the progression of these cancers.

#### 5.2.4 Other cancers

In addition to the abovementioned cancers, emerging evidence has unveiled the aberration and oncogenic potential of JunB in diverse malignancies, including hepatocellular carcinoma (Zhang et al., 2021), oral squamous cell carcinoma (OSCC) (Tsunoda et al., 2021), head and neck squamous cell carcinoma (HNSCC)(Hyakusoku et al., 2016), epidermal neoplasia (Jin et al., 2011), ovarian cancer(OC) (Teng and Zheng, 2017) and lung cancer (Wanna-udom et al., 2020). For example, JunB has been identified as an overexpressed member of AP-1 transcription factor in OSCC. Subsequent investigation indicated that the c-Jun/JunB heterodimer promotes IL-8 transcription in human OSCC derived cell lines (Tsunoda et al., 2021), suggesting the oncogenic role of JunB may be attributed to the formation of complexes with other transcription factors, such as c-Jun. In HNSCC, knockout or knockdown of JunB could inhibit tumor cell migration and invasion *in vitro*, and repress distant lung metastasis *in vivo*, suggesting JunB may be an oncogene in HNSCC progression, but more studies are necessary to be performed to support this point (Hyakusoku et al., 2016). Teng et al. found that JunB was a target of miR-1908, and high expression of JunB correlated with poor prognosis of OC patients (Teng and Zheng, 2017). However, the underlying mechanisms of JunB in genesis of OC need to be clarified. Recently, it was found that both JunB and c-Jun were upregulated in TGF-β1 treated lung cancer cell lines, A549 and LC2/ad. Further studies indicated that METTL3 m6A methyltransferase regulates JunB mRNA and c-Jun protein expression at 3’-UTR in TGF-β1 induced EMT in lung cancer, respectively (Wanna-udom et al., 2020; Suphakhong et al., 2022). This intriguing result was attributed to the fact that different RNA readers bind c-Jun and JunB, YTHDF3 binds c-Jun, and IGF2BP1 binds JunB (Suphakhong et al., 2022). Considering the essential role of JunB in Th2 cells-mediated cytokines production during allergic asthma, further exploration is warranted to determine whether it also exerts regulatory functions within the microenvironment of lung cancer.

## 6 Conclusion and future prospects

In this review, we have systematically elaborated the crucial role of JunB in physiological process and its immune regulatory role (**Table 1**), while highlighting its dual involvement in tumorigenesis (**Table 2**). In addition to modulating tumor cell proliferation, senescence, metastasis, and metabolism, an increasing body of evidence suggests that JunB exhibits significant potential in regulating the TME; conversely, the expression levels of JunB are also influenced by the stroma within the TME. It is widely acknowledged that inflammation plays a pivotal role in cancer development and response to therapy. Specially, JunB has a strong regulatory effect on both innate and adaptive immunity, particularly on immune cell fate determination, differentiation and secretion of inflammatory cytokines. Aberrant expression of JunB is frequently observed in autoimmune diseases, wherein it may contribute to the enhancement of Th cell differentiation and the release of pro-inflammatory factors. Nevertheless, JunB also plays a crucial role in maintaining immune homeostasis under normal physiological conditions by regulating Treg differentiation, owing to its immunosuppressive effects. However, this immunosuppressive effect of JunB can facilitate immune evasion by tumor cells within the TME. JunB deficiency leads to the development of acute and chronic myeloid leukemia, distant metastasis in breast cancer, and enhanced angiogenesis in prostate cancer by modulating the activity of various immune cells, such as promoting pro-angiogenic factor production by neutrophils. Intriguingly, aberrantly expressed JunB facilitates the progression of ccRCC, multiple myeloma, and several lymphomas through transcriptionally promoting the production of chemokines, inflammatory cytokines, and growth factors within the TME. The involvement of JunB in the tumor microenvironment (TME) of these malignancies partially aligns with its role in immune regulation. However, current studies have overlooked the potential regulatory impact of JunB on CAFs, despite their significant influence on TME remodeling. Furthermore, the effect of JunB on the infiltration of T cells and NK cells within the TME remains unexplored. Additionally, the modulation of JunB levels in the TME through post-transcriptional modification and subsequent transcriptional activity on metabolic genes represents a compelling area of interest. Therefore, comprehensive investigations are still required to elucidate the reciprocal regulation between JunB and TME.

**Table 1:** The role of JunB in different immune cells.

Cell Type	Role	Cytokine	Reference
DCs	Promote cytokine production and CD8α cDC1 differentiation	IL-6, IL-12 and TNF-α	(Gomard et al., 2010; Novoszel et al., 2021)
Macrophage	Promote activation and polarization of macrophage	TNF-α and IL-1β	(Fontana et al., 2015a; Fontana et al., 2015b)
Neutrophils	Drive the effector reaction of neutrophils, including promoting the production of ROS, bacterial killing and NET formation	–	(Khoyratty et al., 2021)
NK cells	Contribute to decreasing the killing ability of NK cells	–	(Nausch et al., 2006)
	Promote transcription of IFN-γ	IFN-γ	(Thomsen et al., 2013)
Th2 cells	Contribute to Th2 cell differentiation	IL-4, IL-5, IL-10 and IL-13	(Li et al., 1999; Hartenstein et al., 2002; Chen et al., 2018)
Treg cells	Promote Foxp3 expression in cooperation with c-Rel	–	(Son et al., 2011)
	Promote IRF4 targeting genes expression	–	(Koizumi et al., 2018)
	Promote Treg differentiation by enhancing IL-2 signaling	IL-2	(Katagiri et al., 2019)
	Enhance Treg-mediated immune tolerance	–	(Wu et al., 2019)
Th17	Promote Th17 differentiation and IL-23-dependent pathogenicity of Th17	IL-17A	(Carr et al., 2017; Hasan et al., 2017)
Th 9	Promote IL-9 differentiation in cooperation with BATF and IRF4	IL-9	(Fu et al., 2019)

"-", not available.

**Table 2:** The role and mechanism of JunB in diverse cancers.

Cancer types	Cell or animals	Up stream	Down stream	Mechanism	Role	Reference
AML	NOD-SCID mice	PU.1	NA	Inhibit the self-renewal ability of HSCs and improve AML induced by PU.1 knockout	Tumor suppressor	(Steidl et al., 2006)
	Nr4a3-/-Nr4a1-/- mice	Nr4a3 and Nr4a1	NA	Inhibit the development of AML	Tumor suppressor	(Mullican et al., 2007)
APL	NB4 cells	Skp2	NA	Contribute to the amelioration of APL	Tumor suppressor	(Dan et al., 2022)
T-ALL	Molt 4 and Jurkat cells	miR-149*	NA	Inhibit T-ALL progression	Tumor suppressor	(Fan et al., 2016)
CML	JunB^-/-^ Ubi-junB mice and K562 cells	NA	NA	Inhibiting proliferation and promoting apoptosis of granulocyte progenitors	Tumor suppressor	(Passegue et al., 2001; Yang et al., 2003)
Breast cancer	Junb^Δ/Δ^Col1α2Cre mice	NA	BV8 and MMP9	Inhibit distant metastasis by influencing the initial metastatic stage	Tumor suppressor	(Wutschka et al., 2021)
Prostate cancer	transient amplifying cells (TAC)/DU145; JunB and PTEN double mutant mice in the prostate epithelium	NA	P16 and P21	Contribute to cell senescence that impedes malignant transformation of TAC	Tumor suppressor	(Konishi et al., 2008; Thomsen et al., 2015)
	PCa cells and THP-1 cells	miR-95	NA	Inhibit PCa cell proliferation, invasion, and EMT	Tumor suppressor	(Guan et al., 2020)
ccRCC	786-O and A498 VHL-null ccRCC cells	VHL	MMP2, MMP9 and CCL2	Promote tumor growth and angiogenesis	Oncogene	(Kanno et al., 2012)
MM	Multiple MM cell lines and primary cells	IL-6	VEGF and IGF1	Promote cell survival, proliferation and drug resistance	Oncogene	(Fan et al., 2017; Fan et al., 2021)
HL and ALCL	HRS cell lines ALCL cell lines	CD30 Ets-1 miR-939	ERK1/2-MAPK, and PDGFRB	Promote cell proliferation and metastasis	Oncogene	(Mathas et al., 2002; Watanabe et al., 2005; Watanabe et al., 2008; Laimer et al., 2012; Watanabe et al., 2012; Zhang et al., 2018; Garbin et al., 2021)
OSCC	Ca9‐22 and HSC3	MG132	IL-8	Promote IL-8 transcription	Oncogene	(Tsunoda et al., 2021)
OC	NA	miR-1908	NA	Lead to poor prognosis	Oncogene	(Teng and Zheng, 2017)
Lung cancer	A549 and LC2/ad cell	METTL3	NA	Promote EMT of lung cancer cells	Oncogene	(Wanna-udom et al., 2020; Suphakhong et al., 2022)

NA, not available.

## Author contributions

F-JR and X-YC drafted the manuscript. YY checked the figures and tables and revised the manuscript. G-YF conceived the work and provided the constructive suggestions on the manuscript. All authors contributed to the article and approved the submitted version.
